# Effect of *In Vitro* Testing Parameters on Ceftazidime-Avibactam Minimum Inhibitory Concentrations

**DOI:** 10.1155/2015/489547

**Published:** 2015-05-19

**Authors:** Tiffany R. Keepers, Marcela Gomez, Donald Biek, Ian Critchley, Kevin M. Krause

**Affiliations:** Cerexa, Inc., Oakland, CA 94612, USA

## Abstract

Effects of varying *in vitro* susceptibility testing parameters of the broth microdilution assay on ceftazidime-avibactam MICs were determined and compared to meropenem and piperacillin-tazobactam for 9 *Enterobacteriaceae* and 4 *Pseudomonas aeruginosa* isolates. The effect of varying incubation conditions (ambient air or 5% CO_2_), pH of medium, medium composition (cation-adjusted Mueller Hinton Broth with and without laked horse blood and Haemophilus Test Medium), cation content of the medium, and inoculum density were tested. Most variations had no effect on ceftazidime-avibactam MIC values (no more than a 2-fold change). However, acidic pH or high inoculum resulted in 4- to 16-fold changes in MIC, which was similar to those observed for meropenem and piperacillin-tazobactam under these conditions. Overall, this study shows that slight variations in testing parameters during routine MIC testing will likely have no significant effect on ceftazidime-avibactam MIC values.

## 1. Introduction

Avibactam is a non-*β*-lactam-*β*-lactamase inhibitor with activity against class C enzymes, most class A enzymes (including KPCs), and some class D (OXA) carbapenemases [1]. When ceftazidime is combined with avibactam, the spectrum of activity is expanded to include organisms producing these *β*-lactamases [2–4]. This combination is currently in Phase 3 clinical trials for the treatment of Gram-negative infections.

Susceptibility testing conditions can vary slightly between laboratories and between laboratory scientists. It is important to know if small variations from standard Clinical Laboratory Standards Institute (CLSI) testing guidelines cause any significant changes in testing outcome. These variations can have effects on the MIC when testing *β*-lactams, including cephalosporins and *β*-lactam-*β*-lactamase inhibitor combinations [5–7]. The effect of varying the broth microdilution* in vitro* testing parameters on the ceftazidime-avibactam MIC of *β*-lactamase positive and negative* Enterobacteriaceae *and* Pseudomonas aeruginosa* isolates was determined and compared with the effects on meropenem and piperacillin-tazobactam MICs.

## 2. Materials and Methods

Isolates were obtained from ATCC (Manassas, VA), JMI Laboratories (North Liberty, IA), or the Cerexa, Inc. culture collection. MICs were determined for ceftazidime-avibactam (ceftazidime: USP, avibactam: Forest Laboratories, Inc.), meropenem (USP), and piperacillin-tazobactam (piperacillin: Sigma, tazobactam: USP) by broth microdilution assay according to CLSI guidelines [8, 9]. The effects of the following parameters on the MIC were determined: medium pH 5, 6, and 8 (standard pH 7.2–7.4); incubation with 5% CO_2_ (standard incubation ambient air); the addition of 2.5% laked horse blood (LHB) to cation-adjusted Mueller Hinton broth (CAMHB) and testing in Haemophilus Test Medium (HTM) (standard medium CAMHB); testing in non-cation-adjusted media (MHB without added cations containing 2.9–5.9 mg/L calcium and 3.2–5.2 mg/L magnesium) and testing in 50 mg/L calcium in MHB (standard cation concentration 20–25 mg/L calcium and 10–12.5 mg/L magnesium); and inoculum densities of 5 × 10^4^ CFU/mL and 5 × 10^6^ CFU/mL (standard density 5 × 10^5^ CFU/mL). MIC tests were performed in 2 separate experiments. Data tables list the highest MIC value recorded. Human serum and human serum albumin were previously shown to have no effect on ceftazidime-avibactam MICs [10].

## 3. Results and Discussion

### 3.1. Media pH

In medium with acidic pH (pH 5 or 6), MICs for ceftazidime-avibactam were increased by 4- to 16-fold for 6 of 9* Enterobacteriaceae* isolates and were decreased by 4-fold for 2 of 4* P. aeruginosa* isolates (Table 1). Acidic media caused increased piperacillin-tazobactam MICs against 4 isolates and decreased MICs against 3 isolates by ≥4-fold (Table 2). Four isolates had 4- to >32-fold decreased piperacillin-tazobactam MICs when tested using medium at pH 8. Acidic media caused increased meropenem MICs against 4 isolates and decreased meropenem MICs against 3 isolates by 4- to 8-fold (Table 3). MIC increases were observed at pH 5 against the reference strain* E. coli* ATCC 25922 (*β*-lactamase negative). Thus, the pH effect on MIC appears to be related to the intrinsic antibacterial activity of the *β*-lactams, rather than having an effect on the inhibition of *β*-lactamases.

### 3.2. Incubation Conditions

The ceftazidime-avibactam MIC for one* E. coli* isolate was increased by 4-fold when incubated in 5% CO_2_ (Table 1). Incubation in 5% CO_2_ caused the piperacillin-tazobactam MICs against 2* P. aeruginosa* isolates to change by ≥4-fold (Table 2). The meropenem MICs for all isolates were unaffected (Table 3).

### 3.3. Media Composition

Testing in HTM had no effect on MICs with any drug except for one isolate that had 4-fold increased ceftazidime-avibactam MIC (Table 1). Addition of 2.5% LHB had no effect on ceftazidime-avibactam MICs (Table 1), caused a 4-fold decreased piperacillin-tazobactam MIC against one isolate (Table 2), and caused 8-fold and >32-fold increased meropenem MICs for 2 isolates (Table 3).

### 3.4. Calcium Content

Ceftazidime-avibactam and piperacillin-tazobactam MICs were unaffected by changes in calcium concentration of media (Tables 1 and 2). In medium with trace calcium (MHB), MICs of meropenem decreased by ≥4-fold against one isolate and increased by >4-fold against another (Table 3).

### 3.5. Inoculum

Three isolates had ceftazidime-avibactam MICs that were increased by 4- to 8-fold when the inoculum was increased 10-fold (5 × 10^6^ CFU/mL) (Table 1). Increased inoculum raised the MICs 4- to 16-fold for piperacillin-tazobactam against 3 isolates, and lower inoculum (5 × 10^4^ CFU/mL) resulted in a 4-fold decreased MIC for one isolate (Table 2). High inoculum density caused meropenem MICs to increase by 4- to ≥64-fold for 7 isolates, while low inoculum density caused meropenem MICs to decrease by 4- to 8-fold for 2 isolates (Table 3).

## 4. Conclusions

In summary, most of the variations in broth microdilution parameters had little effect on ceftazidime-avibactam MICs. The two parameters that did have an effect were acidic media (6 isolates) and high inoculum density (3 isolates). Changes in MIC for these conditions ranged from 4- to 16-fold. Other changes to testing parameters only affected ceftazidime-avibactam MICs for a single isolate by only 4-fold in each instance. It is possible that these individual cases are due to random variation rather than reproducible effects on MIC. Piperacillin-tazobactam and meropenem MICs were also affected by changes in pH and inoculum density indicating that these effects are not unique to the ceftazidime-avibactam combination. The magnitude of the changes in piperacillin-tazobactam MICs was more difficult to assess due to the high MICs of most isolates under standard testing conditions.

The inoculum effects noted in this study with ceftazidime-avibactam MICs are not surprising given that inoculum effects have been previously documented with ceftazidime MIC testing, particularly against *β*-lactamase producing strains [5–7]. An inoculum effect on other cephalosporin and carbapenem MICs has also been previously documented; several studies have shown that MICs for piperacillin-tazobactam, and to a lesser extent meropenem, are increased with higher inocula of *β*-lactamase producing and nonproducing isolates [6, 7, 11–13].

These findings indicate that slight variations in testing parameters during routine MIC testing will likely have no significant effect on ceftazidime-avibactam MIC values; however, special attention should be paid to closely follow CLSI guidelines with respect to the pH of the media and the inoculum used. Since most laboratories use commercially prepared media, pH should not be an issue due to the buffering capacity of these media. Additionally, CLSI recommends validating inoculum density on a weekly basis as part of standard quality control so any variations in inoculum should be easily identified [8]. Therefore, if CLSI guidelines and quality control recommendations are followed, there should not be any issues with routine ceftazidime-avibactam MIC testing.

## Figures and Tables

**Table 1:** Ceftazidime-avibactam MICs with variable testing parameters.

Organism	Isolate #	Genotype	Ceftazidime-avibactam MIC (*µ*g/mL)										
			Standard	5 × 10^4^ CFU/mL	5 × 10^6^ CFU/mL	pH 5	pH 6	pH 8	CAMHB + 2.5% LHB	HTM	Trace calcium	50 mg/L calcium	CO_2_
*E. coli *	ATCC 25922	WT	0.5	0.25	0.5	**2**	1	0.25	0.5	0.5	0.25	0.5	0.25
*E. coli *	CML2255	CTX-M-15, TEM-1, OXA-1	0.125	0.06	**0.5**	**0.5**	0.25	0.25	0.125	0.25	0.25	0.25	**0.5**
*E. coli *	CML1468	KPC	0.5	0.5	**4**	**2**	1	1	1	1	0.5	0.5	0.5
*K. pneumoniae *	ATCC 700603	SHV-18	1	1	2	**4**	**4**	1	1	1	1	1	1
*K. pneumoniae *	CML138	CTX-M-15	1	1	1	1	2	0.5	1	1	1	1	1
*K. pneumoniae *	CML128	KPC-2, SHV-27, TEM-1	1	1	2	**4**	**4**	1	0.5	**4**	1	1	2
*K. pneumoniae *	CML148	ΔKPC-2, SHV-27, TEM-1	0.25	0.25	0.5	**4**	**1**	0.5	0.125	0.25	0.25	0.25	0.25
*K. pneumoniae *	CML2280	CTX-M-15, TEM-1, OXA-1	4	2	4	2	4	2	2	2	4	2	2
*K. oxytoca *	CML2251	TEM-129	1	1	1	1	2	1	1	1	1	1	2
*P. aeruginosa *	ATCC 27853	WT	2	2	4	2	2	2	2	2	2	2	2
*P. aeruginosa *	CML1039	AmpC	8	4	16	**2**	8	4	8	8	8	4	16
*P. aeruginosa *	CML1040	AmpC, TEM-24	2	2	4	4	4	2	2	2	2	4	4
*P. aeruginosa *	CML1047	AmpC	8	4	**>16**	**2**	8	8	8	4	4	8	8

MICs obtained under standard CLSI conditions are indicated by the “standard” column header. Parameters that were varied include inoculum density (5 × 10^4^ CFU/mL and 5 × 10^6^ CFU/mL), pH of medium (pH 5, 6, and 8), medium supplements (2.5% laked horse blood (LHB) and HTM), cation content (trace amounts of calcium and 50 mg/L calcium), and incubation with 5% CO_2_. Bolded values indicate a change in MIC of ≥4-fold.

**Table 2:** Piperacillin-tazobactam MICs with variable testing parameters.

Organism	Isolate #	Genotype	Piperacillin-tazobactam MIC (*µ*g/mL)										
			Standard	5 × 10^4^ CFU/mL	5 × 10^6^ CFU/mL	pH 5	pH 6	pH 8	CAMHB + 2.5% LHB	HTM	Trace calcium	50 mg/L calcium	CO_2_
*E. coli *	ATCC 25922	WT	2	2	4	**8**	4	2	2	2	2	2	4
*E. coli *	CML2255	CTX-M-15, TEM-1, OXA-1	32	16	**512**	64	**128**	16	16	64	32	32	64
*E. coli *	CML1468	KPC	>512	512	>512	256	>512	512	512	>512	>512	>512	>512
*K. pneumoniae *	ATCC 700603	SHV-18	16	8	16	**64**	**64**	8	16	16	16	16	8
*K. pneumoniae *	CML138	CTX-M-15	>512	>512	>512	>512	>512	**16**	>512	>512	>512	>512	>512
*K. pneumoniae *	CML128	KPC-2, SHV-27, TEM-1	>512	>512	>512	>512	>512	>512	>512	>512	>512	>512	>512
*K. pneumoniae *	CML148	ΔKPC-2, SHV-27, TEM-1	256	**64**	**>512**	256	256	**16**	**64**	256	128	128	256
*K. pneumoniae *	CML2280	CTX-M-15, TEM-1, OXA-1	>512	256	>512	>512	>512	**32**	>512	>512	>512	>512	>512
*K. oxytoca *	CML2251	TEM-129	>512	>512	>512	512	>512	**256**	>512	>512	>512	>512	>512
*P. aeruginosa *	ATCC 27853	WT	4	4	8	8	4	4	2	2	2	4	4
*P. aeruginosa *	CML1039	AmpC	512	512	>512	**128**	512	>512	512	512	512	512	512
*P. aeruginosa *	CML1040	AmpC, TEM-24	64	64	**256**	**256**	**256**	32	64	64	64	128	**256**
*P. aeruginosa *	CML1047	AmpC	>512	512	>512	**128**	512	>512	512	512	>512	>512	**256**

MICs obtained under standard CLSI conditions are indicated by the “standard” column header. Parameters that were varied include inoculum density (5 × 10^4^ CFU/mL and 5 × 10^6^ CFU/mL), pH of medium (pH 5, 6, and 8), medium supplements (2.5% laked horse blood (LHB) and HTM), cation content (trace amounts of calcium and 50 mg/L calcium), and incubation with 5% CO_2_. Bolded values indicate a change in MIC of ≥4-fold.

**Table 3:** Meropenem MICs with variable testing parameters.

Organism	Isolate #	Genotype	Meropenem MIC (*µ*g/mL)										
			Standard	5 × 10^4^ CFU/mL	5 × 10^6^ CFU/mL	pH 5	pH 6	pH 8	CAMHB + 2.5% LHB	HTM	Trace calcium	50 mg/L calcium	CO_2_
*E. coli *	ATCC 25922	WT	0.016	0.016	**0.125**	**0.125**	**0.06**	0.03	0.03	0.03	0.016	0.03	0.03
*E. coli *	CML2255	CTX-M-15, TEM-1, OXA-1	≤0.06	≤0.06	**0.25**	0.125	≤0.06	≤0.06	≤0.06	≤0.06	≤0.06	≤0.06	≤0.06
*E. coli *	CML1468	KPC	4	**1**	**>8**	**1**	4	4	4	8	2	4	4
*K. pneumoniae *	ATCC 700603	SHV-18	0.03	0.016	0.06	0.06	0.03	0.06	**0.25**	0.03	0.016	0.016	0.03
*K. pneumoniae *	CML138	CTX-M-15	≤0.06	≤0.06	**2**	0.125	0.125	≤0.06	≤0.06	≤0.06	≤0.06	≤0.06	≤0.06
*K. pneumoniae *	CML128	KPC-2, SHV-27, TEM-1	>64	**32**	>64	64	>64	>64	64	>64	**32**	64	64
*K. pneumoniae *	CML148	ΔKPC-2, SHV-27, TEM-1	0.25	0.25	**4**	0.25	0.25	0.5	0.25	0.25	0.125	0.25	0.125
*K. pneumoniae *	CML2280	CTX-M-15, TEM-1, OXA-1	0.06	0.03	**0.5**	0.125	0.06	0.06	0.06	0.06	0.06	0.125	0.06
*K. oxytoca *	CML2251	TEM-129	≤0.06	≤0.06	**4**	0.125	≤0.06	≤0.06	**2**	≤0.06	**0.25**	**0.5**	≤0.06
*P. aeruginosa *	ATCC 27853	WT	0.5	0.5	0.5	0.5	0.25	0.5	0.5	0.5	0.5	0.25	0.25
*P. aeruginosa *	CML1039	AmpC	64	32	64	**8**	32	64	32	32	32	32	32
*P. aeruginosa *	CML1040	AmpC, TEM-24	4	4	8	4	4	8	4	4	4	8	8
*P. aeruginosa *	CML1047	AmpC	16	16	16	**4**	**4**	32	16	16	8	16	8

MICs obtained under standard CLSI conditions are indicated by the “standard” column header. Parameters that were varied include inoculum density (5 × 10^4^ CFU/mL and 5 × 10^6^ CFU/mL), pH of medium (pH 5, 6, and 8), medium supplements (2.5% laked horse blood (LHB) and HTM), cation content (trace amounts of calcium and 50 mg/L calcium), and incubation with 5% CO_2_. Bolded values indicate a change in MIC of ≥4-fold.

## References

[1] Lagacé-Wiens P., Walkty A., Karlowsky J. A. (2014). Ceftazidime-avibactam: an evidence-based review of its pharmacology and potential use in the treatment of Gram-negative bacterial infections. *Core Evidence*.

[2] Castanheira M., Farrell S. E., Krause K. M., Jones R. N., Sader H. S. (2014). Contemporary diversity of *β*-lactamases among enterobacteriaceae in the nine U.S. census regions and ceftazidime-avibactam activity tested against isolates producing the most prevalent *β*-lactamase groups. *Antimicrobial Agents and Chemotherapy*.

[3] Flamm R. K., Stone G. G., Sader H. S., Jones R. N., Nichols W. W. (2014). Avibactam reverts the ceftazidime MIC90 of European Gram-negative bacterial clinical isolates to the epidemiological cut-off value. *Journal of Chemotherapy*.

[4] Sader H. S., Castanheira M., Flamm R. K., Farrell D. J., Jones R. N. (2014). Antimicrobial activity of ceftazidime-avibactam against gram-negative organisms collected from U.S. medical centers in 2012. *Antimicrobial Agents and Chemotherapy*.

[5] Kang C.-I., Cha M. K., Kim S. H. (2014). Extended-spectrum cephalosporins and the inoculum effect in tests with CTX-M-type extended-spectrum *β*-lactamase-producing *Escherichia coli*: potential clinical implications of the revised CLSI interpretive criteria. *International Journal of Antimicrobial Agents*.

[6] Segatore B., Setacci D., Perilli M. (2004). Antimicrobial susceptibility of clinical isolates of Enterobacteriaceae producing complex *β*-lactamase patterns including extended-spectrum enzymes. *International Journal of Antimicrobial Agents*.

[7] Thomson K. S., Moland E. S. (2001). Cefepime, piperacillin-tazobactam, and the inoculum effect in tests with extended-spectrum beta-lactamase-producing *Enterobacteriaceae*. *Antimicrobial Agents and Chemotherapy*.

[8] CLSI (2013). Performance standards for antimicrobial susceptibility testing; twenty-third informational supplement. *CLSI Document*.

[9] CLSI (2012). *Methods for Dilution Antimicrobial Susceptibility Tests for Bacteria That Grow Aerobically; Approved Standard*.

[10] Keepers T. R., Gomez M., Celeri C., Nichols W. W., Krause K. M. (2014). Bactericidal activity, absence of serum effect, and time-kill kinetics of ceftazidime-avibactam against *β*-lactamase-producing enterobacteriaceae and *Pseudomonas aeruginosa*. *Antimicrobial Agents and Chemotherapy*.

[11] Harada Y., Morinaga Y., Kaku N. (2014). *In vitro* and *in vivo* activities of piperacillin-tazobactam and meropenem at different inoculum sizes of ESBL-producing *Klebsiella pneumoniae*. *Clinical Microbiology and Infection*.

[12] López-Cerero L., Picón E., Morillo C. (2010). Comparative assessment of inoculum effects on the antimicrobial activity of amoxycillin-clavulanate and piperacillin-tazobactam with extended-spectrum *β*-lactamase-producing and extended-spectrum *β*-lactamase-non-producing *Escherichia coli* isolates. *Clinical Microbiology and Infection*.

[13] Tam V. H., Ledesma K. R., Chang K. T., Wang T. Y., Quinn J. P. (2009). Killing of Escherichia coli by *β*-lactams at different inocula. *Diagnostic Microbiology and Infectious Disease*.

